# Chemical Sensors and Biosensors in Italy: A Review of the 2015 Literature

**DOI:** 10.3390/s17040868

**Published:** 2017-04-14

**Authors:** Dario Compagnone, Girolamo Di Francia, Corrado Di Natale, Giovanni Neri, Renato Seeber, Antonella Tajani

**Affiliations:** 1 Faculty of Bioscience and Technology for Food, Agriculture and Environment, University of Teramo, 64100 Teramo, Italy; dcompagnone@unite.it; 2 ENEA Italian National Agency for New Technologies, Energy and Sustainable Economic Development, P.le E. Fermi 1, Napoli 80055, Italy; girolamo.difrancia@enea.it; 3 Department of Electronic Engineering, University of Rome Tor Vergata, 00133 Roma, Italy; 4 Department of Engineering, University of Messina, 98166 Messina, Italy; gneri@unime.it; 5 Department of Chemical and Geological Sciences, University of Modena and Reggio Emilia, 41125 Modena, Italy; renato.seeber@unimore.it; 6 Department of Physical Science and Technologies of Matter, National Research Council, 00133 Roma, Italy; antonella.tajani@cnr.it

**Keywords:** chemical sensors, biosensors

## Abstract

The contributions of Italian researchers to sensor research in 2015 is reviewed. The analysis of the activities in one year allows one to obtain a snapshot of the Italian scenario capturing the main directions of the research activities. Furthermore, the distance of more than one year makes meaningful the bibliometric analysis of the reviewed papers. The review shows a research community distributed among different scientific disciplines, from chemistry, physics, engineering, and material science, with a strong interest in collaborative works.

## 1. Introduction

Chemical sensors are devices that convert the concentration of target compounds into an “analytical” signal. The term analytical implies the concept of measurability. Then a chemical sensor converts the information about the presence of target compounds into a measurable quantity.

Currently, electronics is the technology that enables not only the measurement, but also the efficient use of the acquired information. Examples of this are storage, processing, communication, and active utilization of the information to control machines. Sensor technology has been pivotal in the latest pervasiveness of microelectronics, and the continuous extension of sensor properties are gaining unprecedented fields of applications.

Italy has been along the years a fertile ground for chemical sensor development, as witnessed by the constant presence of numerous Italian researchers at the major conferences in the field.

In this paper, a review of one year of scientific results produced in Italy is presented. Of course, this review is far from being an exhaustive description of the potential of all research groups. It is obvious that scientific production may have some discontinuities. Nevertheless, the temporal arc of one year provides a snapshot of the competencies, skills and interest in chemical sensors and biosensors in Italy and may provide a global picture of the situation in this country.

The paper begins with a short presentation of the Italian Association of Sensor and Microsystems that since 1996 has organized a national conference on the wide field of sensors, with a conspicuous presentation of papers on chemical sensors and biosensors. Along the years this conference has become a sort of privileged forum for scientists and technicians involved in the wide field of sensors.

The topics of the paper are then divided into three main categories: gas sensors, electrochemical sensors, and biosensors.

## 2. The Italian Association of Sensors and Microsystems

The Italian Association of Sensors and Microsystems, known by the Italian acronym AISEM, was funded in 1995. The initiative was promoted by Prof. Arnaldo D’Amico as an outcome of the National Project on Sensors, funded by the National Research Council (CNR) within the framework of a large research project on microelectronics. Despite the focus on electronics, the project gathered together researchers in the broad field of sensors. This interdisciplinary approach was imported into AISEM and it is now a distinctive feature of all AISEM initiatives. Among these, the national conference has been most successful. Since the beginning, it was decided that these conferences had to be held in a single session, giving the possibility to all the participants, independently of their own peculiar culture, to attend talks dealing with any sensing topic. This approach is based on the principle that, in spite of the different disciplines, a unified approach to sensor science exists, so that sharing experiences in apparently distant topics is a cultural enrichment. Therefore, at AISEM conferences chemists, physicists, material scientists, electronic engineers and others, share their results and activities, creating unexpected links and fertile research developments.

**Figure 1:**
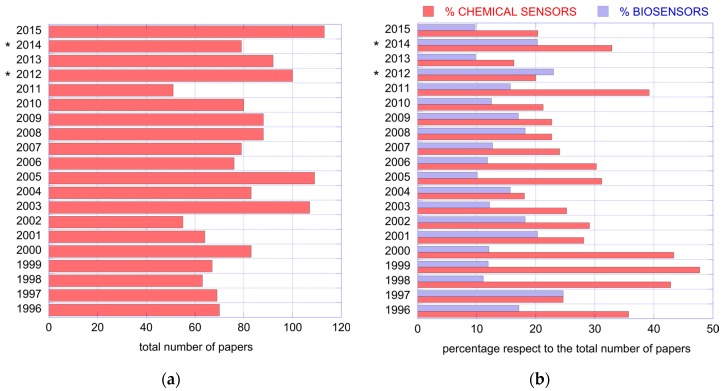
In figure the number of papers published in the proceedings of the Italian conference on sensors and microsystems since the first edition in 1996 is shown. (**a**) total number of published papers; (**b**) percentage of the number of chemical sensors and biosensors papers respect to the total of papers published in the same year. 2012 and 2014 data, marked with a star, are related to the National conference organized by AISEM in cooperation with the major scientific societies interested in sensors.

The first edition of the conference was held in Rome in 1996. The proceedings have been regularly published as edited books. The presence of gas sensors and biosensors has been constant along the years. Figure 1 shows the total number of papers published in the conference proceedings books and the percentage of them dedicated to chemical sensors and biosensors.

In 2015 the conference was held in Trento from the 3rd to the 5th of February and was locally organized by the Fondazione Bruno Kessler and the University of Trento. The proceedings of this edition were published with the technical sponsorship of IEEE [1].

Along the years growing interest in sensor science and technology has prompted some scientific societies to open specific chapters dedicated to sensors. Noteworthy examples are the Società Italiana di Chimica (Italian Chemical Society) and the Società di Ottica e Fotonica (Optics and Photonic Society). For this reason, since 2012 a national conference on sensors was launched with the main purpose of gathering together all the national research societies interested in sensors. Then, after 2012, the national conference alternates with the AISEM conference, which switched to a biannual periodicity. It is worth mentioning, however, that the number of papers in the national conference does not dramatically exceed the number of papers presented at AISEM conferences, evidencing the important role of AISEM on the national scenario.

## 3. Gas Sensors

### 3.1. Gas Sensors Based on Inorganic Materials

The field of gas sensors based on inorganic materials as sensing element was an active area of research in Italy in 2015. Metal oxides are still the most studied inorganic materials for gas sensing, and resistors of these materials are among the most diffused gas sensors [2]. Nanostructured metal oxides in particular attracted most of the interest. These structures are very attractive for gas sensing because of their small size (1–100 nm) and, correspondingly, large surface-to-volume ratio, besides unusual interactions between the target gas and the sensitive element. Changes in particle morphology (shape) as well as in surface reactivity caused by doping with foreign elements, can also dramatically alter the electrical properties of metal oxides when interacting with gaseous species. However, these relationships are complex and not yet well understood at a predictive level, thus experimental studies building connections among the above properties, can provide crucial insights to develop robust strategies improving the performances of metal-oxide gas sensors.

Neri et al. showed the sensitivity to CO and CO_2_ of the resistance of tailor-made ZnO-based materials prepared by the sol-gel technique. In this regard, Ga-doped ZnO sensors exhibited a higher signal and lower response/recovery time than pure ZnO, allowing the detection of CO at sub-ppm concentrations in air [3,4]. Furthermore, the combined effect of Ga doping and UV irradiation allowed monitoring of CO in air at low concentrations with high sensitivity and lower operating temperature [5]. The same authors demonstrated that Ca loading is the key factor in modulating the electrical properties and strongly improving the response of ZnO versus CO_2_. An increased CO_2_ adsorption with Ca loading has been evidenced by FT-IR, providing the basis for the formulation of a plausible mechanism for CO_2_ sensing [6]. The properties of V-doped ZnO:Ca thick films were also studied for ammonia sensing. Results demonstrated the possibility of a fine tuning of the sensing characteristics of ZnO based sensors by Ca and V doping. In particular, their combined effect has brought to an enhanced NH_3_ response compared to bare ZnO and binary V-ZnO and Ca-ZnO sensors [7].

The conductometric responses of sol-gel-based ZnO thin films with embedded 5% mol. Au or Pt nanoparticles (NPs) have been compared at different operating temperatures, to assess the effect of noble metal addition versus H_2_, CO and NO_2_ sensor response [8]. Detection limits as low as 20 ppb NO_2_ and 50 ppm H_2_, in line with the best performances reported for ZnO-based sensors, have been measured. Response times have been also compared, highlighting the positive effects played by Pt addition.

In_2_O_3_-based sensors were demonstrated to have a good response and fast response/recovery times towards very low concentrations of sevoflurane (an anesthetic) in air, suggesting a very attractive application as a real-time monitoring analyzer in a hospital environment [9]. Epifani and coworkers focused their attention on surface modification of TiO_2_ nanocrystals by VO_x_ and WO_x_ coating or wrapping. The comparison of the sensing data for these materials allowed the authors to conclude that the V_2_O_5_ deposition effect could be interpreted as a catalytic contribution, in terms of lowered activation energies of the involved reactions and more favored gas adsorption at lower operating temperatures with respect to pure TiO_2_ [10]. TiO_2_ anatase nanocrystals prepared by solvothermal processing of Ti chloroalkoxide in oleic acid, in the presence of W, with W/Ti nominal atomic concentrations ranging from 0.16 to 0.64, resulted in a three orders of magnitude response improvement with respect to pure TiO_2_ [11].

Novel, non-oxide sensing materials based on chalcogenides, such as CdS and two-dimensional molybdenum disulfide (2D MoS_2_), have been also the subject of some investigations. CdS films show a strong selectivity vs. alcohol, proving that they can efficiently detect alcohols in mixtures where aldehydes and other compounds are present [12]. The unique properties of 2D MoS_2_ have triggered intensive interest due to their intriguing physicochemical properties that stem from a quantum size effect connected with their ultra-thin structure. Donarelli et al. reported a study on the fabrication, the morphological, structural and chemical characterization, and the electrical response to NO_2_ and other gases of resistive type gas sensors based on liquid chemically exfoliated MoS_2_ flakes annealed in air either at 150 °C or at 250 °C [13]. SEM shows that MoS_2_ exfoliated flakes are interconnected between electrodes of the sensing device to form percolation paths. The sensor obtained by thermal annealing in air at 150 °C exhibits a peculiar p-type response under exposure to NO_2_. In line with core level spectroscopy evidences, this behavior is potentially ascribed to nitrogen substitutional doping of S vacancies in the MoS_2_ surface (nitrogen atoms being likely provided by the intercalated liquid, NMP). MoS_2_ flake thermal annealing in air at 250 °C irreversibly imposes an n-type behavior of the gas sensing device, with a NO_2_ detection limit of 20 ppb.

Conductometric gas sensors made of gas permeable metal oxide thin films deposited by physical techniques can combine the functions of a selective filter and sensing element. This can be particularly promising for the sensing of complex mixtures. Gas permeable, ultrathin gas sensing elements can be made suspended enabling advanced architectures of ultrasensitive analytical systems operating at high temperatures and in harsh environments [14]. Ternary thin films of copper aluminum oxide with p-type and n-type behavior made using RF magnetron sputtering for use as chemical gas sensors showed promising results with ozone, acetone and ethanol [15]. With the same technique WO_3_ nanowires were synthesized exhibiting a very good sensitivity, especially for the detection of NH_3_, NO_2_ and CO [16], p-type NiO thin films for detecting low concentrations of ozone (70 ppb) [17] and ZnO films for ethanol vapor sensors, allowing to reach a value of 54 and a limit of detection as low as 610 ppb [18]. Other deposition techniques, such as spray pyrolysis, electrochemical deposition, rheotaxial growth and thermal oxidation (RGTO), laser-enhanced chemical vapour deposition (L-CVD) and so on, have been proposed with the aim to improve the sensing features of thin films metal oxide-based gas sensors [19,20,21,22].

Metal oxide sensors are typically operated at fixed temperature, even if in literature numerous examples of the benefits of temperature modulation in terms of stability and selectivity are reported. An original approach to temperature modulation has been proposed. In this framework the sensor is in a feedback network with the heater. This results in a temperature pattern found to be dependent on the composition of the gas sample. This approach has been demonstrated with a single commercial SnO_2_ sensor applied to the identification of cancer cells and in particular to the discrimination of genetic mutations [23].

Other sensing materials synthesized by the sol-gel method have been also explored for fabricating sensors for different target gases. YCoO_3_ perovskite materials with a tunnel structure and octahedral framework containing platinum or palladium were investigated, analyzing the responses to both oxidizing and reducing gases such as CO, NO_2_, NO and CH_4_ [24].

Surface doping of carbon nanostructured materials (e.g., CNT, graphene) with metal/metal oxide nanoparticles to promote and optimize the sensing performances, represents a research trend in the field of solid-state chemical sensing broadly pursued by Italian researchers. An insight into the nucleation and growth mechanisms involved in the electrodeposition process of Ni nanoparticles has been reported for macroscopic fibers consisting of aligned single wall carbon nanotubes (SWCNT) [25]. An instantaneous nucleation process is responsible for a fast and efficient growth of Ni clusters on SWCNT fiber surfaces, which rules the formation of a hybrid metal-carbon nanostructures material with a high manageability and a potential wide field of application, representing an efficient strategy to produce an active hybrid material with enhanced gas sensing properties. Amorphous titanium dioxide-coated carbon nanotubes (CNTs) were prepared by atomic layer deposition (ALD) and investigated as sensing layers for resistive NO_2_ and O_2_ gas sensors. By varying ALD process conditions and CNT structure, heterostructures with different metal oxide grain size, morphology and coating thickness were synthesized [26]. Higher responses were observed with homogeneous and continuous 5.5 nm thick films onto CNTs at an operating temperature of 150 °C, while CNTs decorated with either discontinuous film or TiO_2_ nanoparticles showed a weak response close to the one of device made of bare CNTs. The feasibility of using polyvinyl alcohol electrospun nanofibers containing Ag nanoparticles for detecting amines by surface enhanced Raman spectroscopy (SERS) after adsorption from the gas phase on the metal NPs, was approached for the detection of biogenic amines [27]. A very simple method was used to completely coat the surfaces of CNTs without requiring any surface modification, creating a thin layer of semiconductor ceramic SiOCN on the CNTs after heat treatment [28]. This new kind of conductometric gas sensor can detect 10 ppm NH_3_ and 2 ppm NO_2_ at temperatures up to 350 °C.

The worldwide explosive interest in graphene research and the development of related applications that followed the Nobel Prize award to Geim and Novoselov in 2010, has also resulted in an exponential increase of the research activity in the field of graphene-based sensors. This resulted in a 10-fold multiplication in the number of the specific research papers in the period 2010–2015 with Italy being in the top ten countries in 2015, in terms of published literature.

As it is well known, the specific material properties of interest for this type of applications are the theoretical very high surface to volume ratio (up to 10,000 m^2^/cm^3^), the minimum permeability to any gas (barrier height up to 8.7 eV) and, finally, the possibility to modulate the electrical properties by means of defect or nanoparticle functionalization engineering. Sensor devices are mostly fabricated using exfoliated graphene or reduced graphene oxide (rGO), sometimes in the form of nanocomposites.

In this respect, a flexible water dispersible chemoresistive gas sensor, fabricated on a nylon-6 membrane via vacuum assisted self-assembly method using a graphene/polystyrene-sulfonate nanocomposite, was investigated for trimethylamine (TMA) detection. The sensor response was found to be repeatable and linear up to a TMA concentration of 183 mg/L with a limit of detection of 23 mg/L [29]. Similarly, a graphene/polyaniline nanocomposite was used as a sensitive layer for gas sensors prepared by dip coating alumina substrates, using a sonication process to assist graphite exfoliation in a water/2-propanol mixture for the graphene nanosheets synthesis. The conductometric device response vs. limonene and ethanol was investigated in various RH concentrations. Interestingly enough it was shown that the relative conductance variation of the nanocomposite vs. RH has an opposite behavior with respect to the pristine PANI [30]. Graphene/ionic liquid (B_mim_^+^Cl^−^ and B_mim_^+^Br^−^) gels were electrochemically synthesized in order to realize nanocomposites characterized by a specific surface area up to 2700 m^2^/g. Both gels have shown a marked reactivity towards caffeic acid, a well-known pharmacological agent, linear in a quite wide range of concentration (from 0.025 to 2.00 M) and with optimal performances in terms of reproducibility (intra reproducibility: 1.40%; inter-electrode reproducibility: 3.20%), sensitivity (3389/μA mM^−1^ cm^−2^), fast response time (2 s) and detection limit (0.005 mM) [31].

As far as graphene oxide is concerned, the change of electrical conductivity of a rGO upon exposure to CO_2_ has been used by Andò and coworkers to realize a flexible chemical sensor on a plastic (PET) substrate with IDE fabricated using an ink-jet technique. The device response, measured at 30 °C, is 45 μΩ/ppm with a limiting sensitivity of 100 ppm, observed to decrease when increasing the device operating temperature [32]. Similarly, a rGO based device, fabricated by drop-casting a solution of GO flakes dispersed in water on a pre-patterned Si_3_N_4_ substrate with 30 μm spaced Pt electrodes, has been studied in various relative humidity environments demonstrating that its response is not affected by the presence of water vapor. The detection limit of NO_2_ was about 20 ppb [33].

Decoration with palladium nanoparticles has been shown to change the electrical properties of graphene upon exposure to different gases. While the pristine p-type material, obtained by the exfoliation of natural graphite in eco-friendly solvents at room temperature, was observed to exhibit an enhanced sensitivity with respect to NO_2_ up to 20 ppb in air at RH = 50%, even quite low concentrations of H_2_ in air (0.2% at RH = 50%) resulted in the formation of palladium hydride nanoparticles that markedly changed the material physical properties. PdHx has in fact a lower work function with respect to graphene so that electrons can flow from the NPs to graphene decreasing its electrical conductivity. As a result, the material became strongly responsive towards hydrogen while the response to NO_2_ faded [34].

### 3.2. Gas Sensors Based on Organic Materials

Organic materials offer a number of undisputable advantages respect to inorganic materials. First of all, the large variety of molecular and polymeric structures that can be synthesized ensure a vast range of sensibility and sensitivity for several applications. Furthermore, organic materials are typically used at room temperature which is a benefit with respect to inorganic materials that are usually operated at high temperature. On the other hand, organic materials are seldom conductive, and then different transduction techniques are typically used in this case. The most practiced are optical and mass transducers.

The use of colorimetric dyes or fluorescent molecules as detectors is a very well-known practice in analytical chemistry. In the last two decades there has been a growing interest in the use of optical devices such as cameras, scanners, and monitors as transducers. This approach enables the development of low-cost applications where ordinary equipment is momentarily used as a sensor.

Porphyrins have been widely used as optical reporters, however the sensitivity of these molecules is limited mainly to Lewis acid-basis compounds. Dini et al. illustrated an original approach to develop optical reporters exploiting the mutual interaction between two different optically active molecules [35]. Blends of porphyrins and pH indicators showed a sensitivity and selectivity that largely exceed that of the individual molecules. An array of combinatorially prepared materials shown to be able to detect a large number of volatile compounds including those usually not sensed with optical sensors, such as hexane.

Mass sensors represent another class of transducers that can be easily used with molecular and polymeric films. Porphyrin-coated quartz microbalances have been used for several years. Arrays of such sensors that change with suitable changes of the porphyrin molecule can be easily obtained. In particular, the sensitivity and selectivity are strongly dependent on the metal ion complexed at the core of the porphyrin aromatic ring. These arrays have been used to retrieve qualitative information in many different fields. A noteworthy example is breath analysis for medical diagnosis. In this regard, Capuano et al. demonstrated the capability of such an array to identify different stages of lung cancer [36].

Capuano et al. focused their attention to the role of the aromatic ring studying two homologous series of metalloporphyrins and metallocorroles [37], these being a porphyrinoid where two of the pyrroles of the aromatic core are directly linked. Results showed that the same metal but on different rings results in a differently behaving sensor. This finding provides an additional degree of freedom for the design of porphyrin-based sensor arrays.

One of the open problems in sensor arrays is the expansion of array selectivity, for which a useful approach consists in using different sensing materials in the same array. Pizzoni et al. complemented porphyrins with short peptides sequences giving rise to a hybrid array of quartz microbalances that has been positively used to characterize the aroma of flavoured gummy candies [38].

It is interesting to mention the sensing properties of natural extracts such as flavonoids. Costa et al. reported the discrimination of white truffles of different qualities using an array of quartz microbalances coated by flavonoid extracts from plants [39]. The characteristics of some of the discussed sensors are summarized in Table 1.

## 4. Electrochemical Sensors

Till the nineties, and even later, the term *electroanalytical chemistry* was used to indicate the development of novel devices and techniques, and of novel methodologies for the correct use of electrochemical techniques in analytical chemistry. It also included all techniques (spectroscopic, morphologic, structural, etc.) suitable for characterising the electrically conducting materials constituting the electrode or the species used as a component of the electrode. The term also included these non-electrochemical techniques whenever used to identify the effects induced by the polarisation of the electrode, such as transformation of species, of the electrode surface itself, etc.

The outstanding Bard’s series (different editors have continued this work in subsequent years) defined what we would like to define as *electroanalytical chemistry* in a wider meaning. It is not clear to us whether the term *electroanalysis* refers to something different.

Most part of the activity of the electroanalysis starts from a more restricted view of Electroanalytical Chemistry, which is in most cases identified with Electrochemical Sensing. Electrochemical Sensing is strictly linked to: (i) realization and physico-chemical characterization of novel materials or molecules acting as the sensitive elements of the sensors; (ii) use of some benchmark species to test the effectiveness of the developed system and of the relevant analytical procedure; (iii) finalization of the newly realized or already established sensing devices to detect specific substances in specific matrices, developing suitable procedures to achieve best values for “performance indicators”. Food or environmental matrices are most widely taken into consideration; those dealing with human health are less dealt with, being more frequently approached by biosensors. The three points listed not always constitute the subject of the work of one single project: some researchers prefer concentrating their efforts on one or two of them, although feedbacks from the different steps of the pipeline are of impressive value.

As to the first identified issue, the extraordinary upsurge of material science has induced electroanalysts to exploit a number of novel, conductive materials either as the sensitive elements of sensors or as a part of composites bearing molecules or functionalities suitable to more or less specifically interact with certain analytes. A minor portion of new materials, actually not necessarily conductive, have been exploited in potentiometry, to realize effective Ion Selective Electrodes (ISEs). Conductimetric sensors have been developed for both liquid and gaseous samples and impedance measurements have been mainly, even if not exclusively, exploited in the frame of biosensing. It is however out of question that electrochemical controlled potential techniques attract nowadays the very major attention by researchers in Italy and worldwide. Actually, the perspectives for potentiometry and for controlled potential techniques could be located in opposite positions: on the one side ISEs have reached a satisfactory effectiveness for a series of compounds and, on the other side, often too high effort-to-“production” ratio may be expected from the sometimes speculative works on tools for voltammetric techniques in academia.

Novel materials have been least frequently used as such; they most often need to be supported on a conductive conventional substrate, such as Pt, Au or glassy carbon, leading to the so-called (chemically) modified electrodes. The role of the modification is essentially that of pursuing electrocatalytic effects and of possibly preventing adsorption of species present in solution that poison the electrode surface. In particular, electrocatalysis may anticipate the potential at which some analytes are electroactive, favoring higher resolution or even allowing detection of species otherwise “hidden” by the solvent discharge [40].

Discussing the approaches followed in order to induce electrocatalysis and prevent electrode fouling, we can work out a concise examination of the most often preferred amperometric system.

In [41] 3D-ensembles of gold nanowires were realized by electroless Au deposition in polycarbonate membranes by partial membrane etching. These electrode systems, characterized by a particularly high signal-to-noise (Faradic-to-capacitive currents) ratio are applied to the anodic stripping voltammetric determination of inorganic arsenic, which constitutes an analyte of particular interest.

For many reasons, among which the fact that excellent repeatability of the responses is not mandatory, and that the cost is very low, outstanding interest has been recently acknowledged to disposable systems consisting of Screen Printed Electrodes (SPEs). A simple carbon black dispersion on a SPE is effectively used for determination of a number of phenolic compounds [42]. The effectiveness of composites is coupled to the advantages of SPEs in a sensing system in which Au-NPs are anchored to carbon black microparticles [43].

A series of modifiers consisting of Layered Double Hydroxide (LDH) films containing transition metals suitable to act as redox mediators is profitably used in electrochemical sensors. Continuing a research line that produced abundant literature from this same group, in [44] redox mediation by the Co(IV)/Co(III) redox couple was exploited for the determination of glucose, fructose, and sucrose at the exit of an HPLC chromatographic system, operating in flux conditions. Within the same line of research, Ni/Al LDH thin films are electrochemically generated onto Pt surfaces.

An interesting class of sensors is that based on the concurrent optical and chemical sensitivities. The combination of porphyrins and ZnO was particularly appealing in the past for dye sensitized solar cells development. Actually, both these materials are also excellent chemical sensors as discussed in the previous section. These materials have also been found suitable as amperometric electrodes. ZnO nanorods coated with either 5,10,15,20-tetra(4-sulphonatophenyl)-porphy-rinato]Mn(III)Cl or [5,10,15,20-tetra(4-sulphonatophenyl)-porphyrinato]Co prepared in one-pot have been used as electrodes for the detection of l-cysteine [45]. Results show that the illumination with visible light, absorbed by porphyrins, elicits and increases the sensitivity and selectivity.

An interesting couple of papers evidenced the possibility to detect analytes beyond the Debye length from the sensor surface. The Debye length is the distance of decay of the electric field from the electrode surface, then in some sense it defines the volume of solution where the sensor is sensitive. The extension of sensitivity beyond the Debye length has been achieved operating an array of capacitive sensors at high frequency [46]. The method was shown to be effective for instance to image living cells. A different paper showed that the Debye length limit can be avoided in Extended Gate Field Effect Transistors where the response to an analyte is governed by a capacitive effect that does not depend on Debye’s length [47].

As an example of performance, Table 2 lists the main features of the some sensors illustrated herewith.

## 5. Biosensors

According to the classical IUPAC definition, a biosensor is an analytical device which is capable of providing specific quantitative or semi quantitative analytical information using a biological recognition element (biochemical receptor) which is in direct spatial contact with a transducer element. In recent years, among the biological elements, different receptors mimicking antibodies have been developed, e.g., aptamers, molecularly imprinted polymers, synthetic peptides. They can be included in the biosensor area. Moreover, the widespread use of nanomaterials and nanostructured surfaces has given a very robust input to the area. In fact, the large surface to volume ratio of nanomaterials offers potential advantages in terms of sensitivity and gives different opportunities for the anchoring of the biological element. This has progressively led to the real integration of the biomolecules into the transducer. Searching in Scopus, for papers on biosensors, published by Italian groups in 2015 resulted in over 90 original papers in international peer reviewed journals and 10 reviews on different applications related to biosensors. Looking at the type of transduction described in these papers, the Italian community like the rest of the world, increasingly used different optical detection configurations, with particular emphasis on newly designed Surface Plasmonic Resonance-based devices for label free detection of target analytes. However, the majority (about 60%) of the realized biosensors employed some kind of electrochemical detection in all the different possible ways, amperometric, voltammetric and impedimetric (for label-free) being the most used. Catalytic biosensors based on immobilized enzymes represent half of the production with respect to affinity sensors for which the antibodies, as binding elements, are mostly used together with proteic receptors, molecularly imprinted polymers and aptamers. A relevant number of works on affinity sensing deal with strategies to improve detection of selected DNA sequences in hybridization reactions. The majority of the research is addressed to the determination of clinically relevant markers and analytes of importance in food quality and safety. Considering the number of papers, this section will not treat them exhaustively; a selected number of significant examples on catalytic and affinity sensors will be given with particular emphasis on the biosensors tested in real samples. In fact, considering the complexity of the device and the potential effects of the samples onto the performance of both the transducer and the biological element, this is very often the critical step for the realization of a successful biosensor.

### 5.1. Catalytic Biosensors

Classical first generation enzyme electrodes were originally developed 50 years ago. The technique of enzyme immobilization onto the transducer surface, resulting in a response mainly dependent on both the diffusion of the target substrate to the enzyme active site and on the product of the reaction to the transducer surface, is still very often used. In fact, the possibility of using different types electrodes, particularly screen-printed carbon-based ones, that can be easily chemically modified with mediators and nanomaterials, is attractive to enhance the sensitivity, and selectivity and reduce the cost of the enzyme electrode. In this respect, Cinti et al. [48] developed a cholesterol biosensor for human serum based on screen-printed electrodes (SPEs) modified with inkjet-printed Prussian Blue nanoparticles (PBNPs). The so called “artificial peroxidase” Prussian Blue, represents a very useful mediator. In this case, real samples are expected to have easily oxidisable compounds since the detection of the hydrogen peroxide, generated by the oxidase enzyme, is obtained via reduction at very low over-potential. This has been also demonstrated by Sannini et al. who, by using a similar approach (SPE modified with Prussian Blue), realized a lactate biosensor able to monitor the malolactic fermentation in an intereferent-rich matrix like wine [49]. Polyamines, such as spermine and spermidine, whose assessment represents an important analytical tool in food analysis and human diagnostics, have been also detected. The technique used newly engineered polyamine oxidase and spermine oxidase, entrapped in a photocrosslinkable gel of poly(vinyl alcohol) bearing styrylpyridinium groups, onto carbon SPE electrodes chemically modified with Prussian Blue [50]. A well-known different strategy to realize interference-free first generation enzyme electrodes is the use of a selective barrier layer on the electrode surface onto which the enzyme can be retained by physical entrapment or cross-linking. Over-oxidised polypirrole is an ideal permselective membrane candidate. This has been further demonstrated in the realization of a lysine enzyme electrode assembled using a Pt electrode and lysine oxidase; the biosensor was used in a very challenging sample as cheese in order to assess maturation [51]. Glucose oxidase-based biosensors for the detection of glucose are the most published biosensors. However novel interesting solutions for new types of glucose sensors can be still realized using glucose oxidase. Two clear examples are represented by the works of Strambini et al. and Habtamu and Ugo [52,53]. The first approach reports the development of a self-powered microneedle-based transdermal biosensor for the real-time measurement of glycaemia in interstitial fluid. The microneedles, having a glucose biosensor integrated on the back-side of the needle-chip, operate under capillary action and are able to measure glucose with high accuracy (±20% of the actual glucose level for 96% of samples) in 30 s. A miniaturized sensor is also reported in the second case: the properties of nanoelectrode ensembles, prepared by electroless gold deposition in track-etched polycarbonate (PC) membranes, have been exploited for the realization of a second generation enzyme electrode. (Ferrocenylmethyl)trimethylammonium was used as the mediator; the detection limit was 36 μM of glucose. First generation enzyme electrodes using NAD(P)-dependent dehydrogenases can also be realized providing that fouling of the electrode surface, due to the oxidation of the cofactor, is somehow prevented. This has been achieved by Malvano et al. [54] using alcohol dehydrogenase immobilized onto a SPE electrode modified with polyaniline doped with poly(2-acrylamido-2-methyl-1-propane) sulfonic acid polymer. Unfouling detection of NADH at 0.1 V vs. Ag/AgCl in the 0.005–2 mM range allowed the determination of ethanol in red and white wine samples. Enzyme electrodes can be also used for the evaluation of functional parameters, due to the antioxidant capacity of food that is given by molecules of different chemical structure present in the sample. Antioxidant capacity, composed by pure reducing, metal chelating and radical scavenging activity is generally tested using different spectrophotometric assays. The performances of a superoxide dismutase enzyme sensor for the antioxidant capacity assay of mixed berries and jams, yoghurts and juices containing mixed berries, were favourably comparable to classical methods as reported by Tomassetti et al. [55].

Dual assessment of ascorbic acid and antioxidant activity in blueberry, kiwi and orange juice was achieved by Barberis et al. [56]. They used a fullerenes- and nanotubes-modified graphite sensing-biosensing setup based on an ascorbate oxidase, coupled with a dual-channel telemetric device. The amount of ascorbate was obtained by differential measurements considering the decrease in current due to the oxidation by the immobilized enzyme. Inhibition-based enzyme electrodes have been mainly reported for the detection of pesticides exploiting the inhibition effect of some carbamates and organophosphates on the enzyme acetylcholinesterase. However, other enzymes such as atrazine, can be used to detect pesticides. Atrazine is, in fact, able to inhibit the activity of the enzyme tyrosinase and an electrode based on this enzyme immobilized onto SPE using different carbon nanomaterials has been proposed for the detection of atrazine in drinking water [57]. Atrazine detection range was within 0.5–20 ppm, with a LOD of 0.3 ppm under the optimized conditions. A peculiar application of bilirubin oxidase enzyme electrode has been reported by Grattieri et al. [58]. The sensor was used to carefully assess oxygen profiles in a membraneless, single-chamber microbial fuel cell. Oxygen concentration in biofilms covering electrodes was accurately measured; the generated power of the microbial fuel cell was found to be in the 0–0.08 mW range and was dependent on oxygen content. The only example of optical detection for catalytic biosensors reports a platform of different enzymes as acetylcholinesterase, tyrosinase, urease, β-galactosidase and d-lactate dehydrogenase enzymes together with C. reinhardtii cells for the safety management of milk. The detection of the enzymatic activities has been achieved using fluorescence signal of fluorescein 5(6)-isothiocyanate or 5(6)-carboxynaphthofluorescein while for the algal cells, fluorescence emission of chlorophyll was used. The LODs obtained with the multiarray of enzymes were 1.1 ng/kg for diuron, 0.6 μg/L for chlorpyrifos, 1.2 μg/L for catechol, 13.8 mg/dL for urea, 0.06 g/L for lactose, and 19.5 ppm for D-lactic acid [59]. The main characteristics of the discussed catalytic biosensors are outlined in Table 3.

### 5.2. Affinity Biosensors

Antibody-based affinity biosensors use antibodies as selective biological element. The research carried out in classical immunoassays in the last 40 years gave a great impulse to the development of immunosensors, particularly for the assay format, optimization of the incubation time, amplification of the signal, immmobilisation and orientation of the antibody. However, the widely used spectrophotometric ELISA format remains very competitive in terms of sensitivity and cost. Thus, the research efforts in the last years have been mainly devoted to the development of improved label- free assays, particularly with optical detection. For example, label-free evanescent wave optical fiber immunosensors can be realized using titania-silica-coated long period gratings [60]. The chemical overlay, which increases the refractive index sensitivity of the sensor, consists of a sol-gel-based titania-silica thin film, deposited along the sensing portion of the fiber by means of the dip-coating technique. Analyte detection was achieved from the wavelength shift at the end of the binding process and from the initial binding rate. The system has been tested for IgG detecting anti-IgG into human serum with detection limit of the order of tens of micrograms per liter. A further example can be represented by the use of micro-pillars, columnar resonators that can be arranged in dense arrays of several thousand sensors in a squared mm, as reported by Tardivo et al. [61]. The authors propose a detection method based on CCD imaging and software image analysis, which can measure the resonance frequency of tens of pillars in parallel. Acquisition of the frequency shift of a series of sensors (40 in this case) overcome the variability of the single sensor measurement. An immunosensor for prostate specific membrane antigen was able to detect the tumor marker in the 0.3–100 nM range. An electrolyte-gated organic field-effect transistor (EGOFET) operated as an immunosensor for interleukin 4, an inflammatory citokyne [62]. Single-molecule force spectroscopy, performed across areas of an immunosensor, allowed the study of the length scales, the adhesion energy, and the time scales of the selective recognition. Immobilization of the antibody using a smooth film of His-tagged protein G on the gold electrode gave the highest probability (30%) of specific-binding events detected by force spectroscopy. It was demonstrated that this type of immobilization gave higher areal density of oriented antibodies available for recognition with respect to a 6-aminohexanethiol + glutaraldehyde coupling procedure. The simplicity and competitiveness of the ELISA assay format can be also exploited for portable detection systems where the detector is a mobile phone. This approach has been reported by Zangheri et al. for the detection of cortisol in human saliva [63]. A chemiluminescent-lateral flow immunoassay method has been integrated in a smartphone for the purpose. A direct competitive assay using peroxidase-cortisol conjugated with luminol/enhancer/hydrogen peroxide as substrates were used. Smartphone camera played a light detector, for image acquisition and data handling via a specific application. Accessories to adapt the smartphone were 3D-printed. A detection limit of 0.3 ng/mL with quantitative analysis in the range of 0.3–60 ng/mL was achieved. This was adequate for detecting salivary cortisol in the clinically accepted range. This approach appeared significant in the growing area of home-self-diagnostic device technology for clinical biomarker monitoring.

Aptamers are single stranded DNA or RNA molecules that have been selected using in vitro techniques to bind target molecules with high affinity and selectivity, rivaling antibodies in many ways. Depending on the affinity of the aptamers and on the technique used, detection limits in the nanomolar range can be achieved in label-free biosensors also for molecules of relatively low molecular weight as, for example, toxins. A voltammetric/impedimetric biosensor for aflatoxin B1 (AFB1), a mycotoxin identified as contaminant in food, was realized by Castillo et al. using differently derivatised dendrimeric structure for the immobilization of an aptamer [64]. 0.1–10 nM concentration range of aflatoxin B1 was determined with a limit of detection of 0.40 nM. The aptamer biosensor was tested in certified contaminated peanuts extract as well as in spiked samples of peanuts based corn snacks with satisfactory results. The M1 form of aflatoxin, a toxin of major concern for the southern Europe dairy industry, was the target of the study of Guider at al. They used a newly developed optical label-free detector consisting in an array of multiple SiON microring resonators, fiber-coupled to both an 850 nm VCSEL and a silicon photodetector, packaged with a microfluidic circuit [65]. Multiple sequential measurements were possible after chemical regeneration of the sensing surface. The limit of detection was, in this case, 1.6 nM. Improved sensitivity and very low detection limits can be achieved with aptamers using strategies similar to classical immunoassays. Scarano et al. realized a piezoelectric biosensor for human matrix metalloproteinase 9, a protein associated with cardiovascular dysfunctions, pathologies of the central nervous system, neuropsychiatric disorders and degenerative diseases related to brain tumors able to work in the picomolar range [66]. Sensitivity was improved using two different aptamers in a sandwich-like approach. Two different aptamers were also used in a “plasmonic” assay for the detection of single methylation of DNA occurred in one of the aptamers by Tintorè et al [67]. Plasmonic detection has been recently referred to any assay that takes advantage of the peculiar plasmonic absorption of gold and other metal nanoparticles. The authors used Au-coated magnetic nanoparticles derivatised with two aptamers for the α-thrombin protein. In the presence of the protein, aggregation of the nanoparticles, causing changes in UV and color of the solution, was observed. Single methylation of one of the aptamers prevented the aggregation, demonstrating the potential use of the biosensor in applications related to DNA repair detection.

A tumor marker, human epidermal growth factor receptor 2 (HER2), was the target of a sensitive label-free impedimetric biosensor developed by Ravalli et al. [68] The authors used in this case an affibody, a protein generated using a combinatorial protein engineering approach for the target analyte. These molecules represent new binders able to stick to different target proteins, retaining the favorable folding and stability properties. By optimizing experimental conditions, a single-use affisensor had a good analytical performance for HER2 detection from 0 to 40 μg/L. The estimated limit of detection was 6.0 μg/L. The ability of human serum albumin and rat serum albumin to bind novel bicalutamide analogues by surface plasmon resonance was exploited by Fortugno et al. [69]. The work demonstrates that SPR-based optical biosensor technique is well suited for the high throughput screening of ligands binding to serum albumins. This behavior highlights possible differences among species when performing pharmacokinetic studies.

Odorant proteins can be used as well for the development of bioelectronic noses to detect target analytes in the gas phase. A remarkable result has been obtained by Mulla et al. who developed an organic transistor whose gate was functionalized with a self-assembled monolayer of an odorant binding proteins [70]. Of note, the sensor showed the capability to separate enantiomers of carvone.

Di Pietrantonio et al. have used laser-induced forward transfer (LIFT) for the deposition of wild-type bovine and porcine odorant proteins and double-mutant bovine odorant-protein onto the active area of surface acoustic wave resonators [71]. The biosensor was able to discriminate octenol molecules from carvone molecules and is proposed for the assessment of food contamination by moulds. Selective detection of butanal was instead achieved by Cennamo et al using porcine odorant-binding protein in connection with surface plasmon resonance transduction in a plastic optical fiber tool [72]. The use of plastic optical fibers allows the use of surface plasmon resonance at low cost an in a “lab-on-a-chip” platform. Butanal was detected in the 20 μM–1000 μM concentration range. Alternatively to odorant binding proteins, short peptide sequences can be also used for the detection of volatiles in a sensor array format. Compagnone et al. detected off-flavours in chocolate samples using an array of quartz crystal microbalances modified with gold nanoparticles bearing short peptide sequences [73]. Running a PLS-DA analysis the peptide array was able to discriminate over 95% of the off-flavoured chocolate samples demonstrating the potential of using short peptides in electronic noses.

Real-time label-free direct electronic monitoring of the activity of the enzyme human DNA topoisomerase IB on graphene was finally shown by Zuccaro and co-workers. By monitoring the field-effect characteristics of the graphene biosensor in real-time during the enzyme-substrate interactions, the surface binding constant for the cleavage reaction step of topoisomerase I activity down to picomolar concentrations was obtained. This is a relevant result for future rapid screening of drugs, being evaluated for regulating enzyme activity. It is worth to observe that this is one of the very few examples of a sensor device fabricated using a CVD monolayer graphene substrate [74].

Detection of DNA hybridization is a powerful diagnostic tool in many different fields of application. Biosensors have great potential for the sensitive detection of the hybridization reaction. However, to achieve the required selectivity and sensitivity a careful selection of the sequence immobilized of the probe and assay condition is necessary. Ultrasensitive human genomic DNA detection, directly extracted from lymphocytes without the PCR amplification step has been recently reported by Mariani et al. [75]. Gold nanostars have been applied in a sandwich-like assay based on the selective capture of specific DNA targets and the subsequent signal amplification by a secondary DNA probe linked to the nanoparticles. A SPR imaging platform was used as detector. The use of short synthetic DNA target sequences together with gold nanoparticles lowered 610-fold the detection limit from 6.1 nM to 10 pM using. A similar decrease of detection limit was obtained on genomic DNA samples, extracted from human lymphocytes from 3 fM to 6.9 aM. Unamplified DNA was also detected by an optofluidic device consisting of microstructured optical fibers containing microchannels and Bragg grating [76]. A peptide nucleic acid probe specific for a gene tract of the genetically modified Roundup Ready soy was used to capture DNA. Enhancement of optical signal due to a shift in the reflected light was, in this case, obtained using streptavidin coated gold-nanoparticles. Grating coupled surface plasmon resonators under azimuthal control of incident light has been proposed for the development of biosensing solutions alternatively to classic prism-coupled SPR sensors. This configuration has the advantage of higher miniaturization potential. Silvestri et al. optimized a PNA-based sensing layer co-immobilising the antifouling agent poly(ethylene oxide) and the PNA probe [77]. By probing the DNA of the tuberculosis mycobacterium, the authors achieved lower detection limit than a fluorescence assay both for the complementary target and for DNA amplicons. Giamblanco et al. have reported the potential use of quartz crystal microbalances for unamplified DNA detection [78]. They proved that selective sensing for a large DNA target, constituted by a clone of 7 kbps, can be obtained through a proper optimization of the probe density on the resonator surface which gave a detection of fmoles per cm^−2^ of target. Electrochemical strategies still have potential for DNA hybridization sensing. In fact, the combination of a sensitive electrochemical platform and a proper microfluidic system using a simple enzyme signal amplification assay has been used by Asl et al. for detection of the target DNA sequence and single nucleotide mutation in p53 tumor suppressor gene sequence [79]. The biosensor was able to discriminate successfully perfect matched DNA from mutant form in the PCR amplified samples.

Table 4 lists the main features of the discussed affinity biosensors.

## 6. Bibliometric Analysis

**Figure 2:**
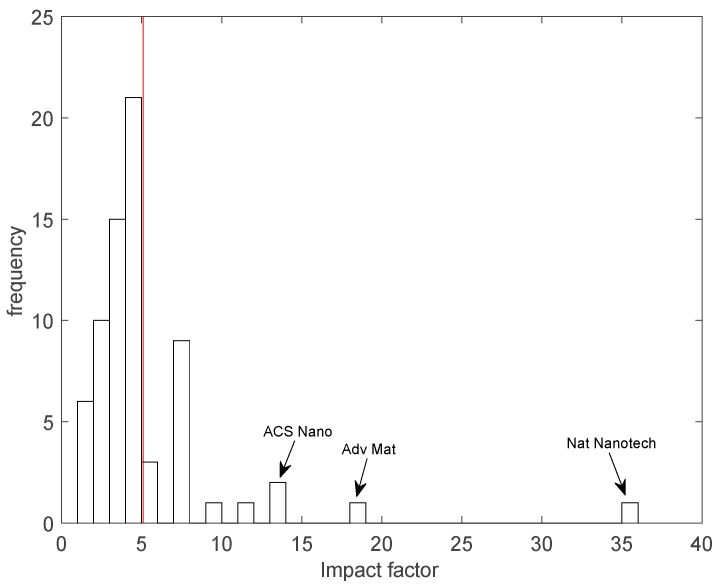
Reviewed papers distributed along the journal impact factor. The journals with the largest impact factor are explicitly indicated. The average impact factor is 5.11 and it is marked by the vertical red line.

The collection of papers reviewed here has been published in 44 different journals. The largest number of papers were published in *Sensors and Actuators B* and *Biosensors and Bioelectronics*, with 13 and seven papers, respectively. Figure 2 shows the histogram of the number of papers distributed according to the 2015 journal impact factorf. These figures were provided by the 2016 Journal Citation Index and retrieved from the webpage of each journal.

The average impact factor is 5.11. The distribution shows a compact behaviour in the range 0–6 where the large number of papers in *Sensors and Actuators B* (IF = 4.758) dominates. After this range the distribution is sparse with a dominating peak due to papers on *Biosensors and Bioelectronics* (IF = 7.476).

Noteworthy papers with the largest impact factors are those in *ACS Nano* (IF = 13.334) published by the groups of Biscarini [62] and Desideri [64], *Advanced Materials* (IF = 18.960) published by the group of Torsi [47], and *Nature Nanotechnology* (IF = 35.367) from the Selmi group [46].

**Figure 3:**
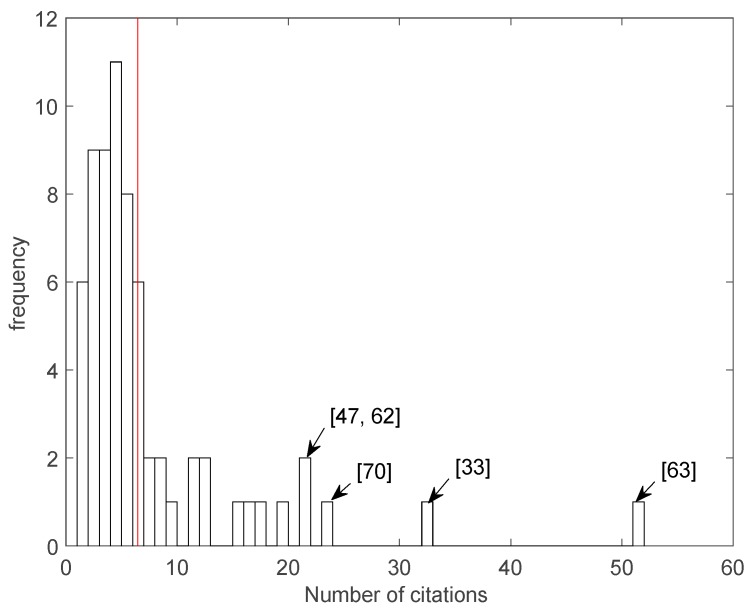
Distribution of the number of citations of the papers here reviewed. The reference of papers cited more than 20 times are shown. The red vertical line marks the average number of citations.

The impact of individual papers has been evaluated considering the number of citations as retrieved from the Scopus database [80]. The average number of citations is 6.4 and only six papers have never been cited so far. Figure 3 shows the distribution of papers according to the number of citations. After 10 citations, the distribution ceases to be continuous. It is worth mention that a paper from the Roda group [63] was cited 51 times.

## 7. Conclusions

Sensor technologies have nowadays a great impact on our daily life. Physical sensors based on micromechanical technology are embedded in familiar devices, such as smartphones, and are enabling the development of machines, such as minidrones, that were unforeseen only a few years ago. Chemical sensing and biosensing have still not reached the maturity to be included in such massively diffused goods, however, there is a wide need for such devices for a large number of applications ranging from medical diagnosis, to environmental control and food safety monitoring. Hence, there is a great research activity on chemical sensors and biosensors and Italian research groups are actively contributing to this development. However, it is important to be aware that the current demand requires a strong interaction between microsystems technology and chemical sensors and biosensors technology. Chances of success are enhanced by common studies in these complementary areas, as demonstrated by many papers here reviewed that stem from collaboration among different groups and sometimes even among researchers of different disciplines.

## Figures and Tables

**Table 1:** Features of the discussed gas sensors.

Sensitive Material	Method	Analyte	Sensors Performance	Ref.
Ga-ZnO	Conductometric	CO	LOD * = 0.8 ppm CO, *R*_0_/*R* = 9 (50 ppm CO) *T* = 250 °C	[3]
ZnO-Ca	Conductometric	CO_2_	*ΔR*_0_/*R* = 113 (5% CO_2_) *T* = 450 °C UV light-assisted	[6]
Ca-V-ZnO	Conductometric	NH_3_	*R*_0_/*R* = 3 (1000 ppm NH_3_) *T* = 250 °C	[7]
Pt-ZnO	Conductometric Optical	NO_2_, H_2_	*LOD* = 20 ppb NO_2_, 50 ppm H_2_ T = 300 °C	[8]
In_2_O_3_	Conductometric	Sevoflurane	*R*_0_/*R* = 5.8 (1.5 ppm Sevoflurane) T = 100 °C	[9]
V_2_O_5_-TiO_2_	Conductometric	Acetone, Ethanol	*R*_0_/*R* = 9 (100 ppm Acetone) T = 300 °C	[10]
WO_x_-TiO_2_	Conductometric	Ethanol	*R*_0_/*R* = 5 (100 ppm Ethanol) T = 200 °C	[11]
CdS	Conductometric	Alcohols	*ΔR*_0_/*R* = 63 (5 ppm Ethanol) T = 300 °C	[12]
MOS_2_	Conductometric	NO_2_	*LOD* = 20 ppb NO_2_ *R*_0_/*R* = 2.7 (1 ppm NO_2_) *T* = 200 °C	[13]
Spinel CuAl_2_O_4_	Conductometric	O_3_	*LOD* ≤ 1 ppb O_3_ *ΔR*_0_/*R* = 100 (70 ppb O_3_) *T* = 300 °C	[15]
WO_3_ nanowire	Conductometric	CO, NH_3_, NO_2_	*LOD* = CO 13 ppm, NO_2_ 1 ppm *T* = 100–200 °C	[16]
NiO thin film	Conductometric	O_3_	*ΔR*_0_/*R* = 12.3 to 70 ppb of O_3_, *T* = 200 °C	[17]
ZnO film	Conductometric	Ethanol	*LOD* = 0.61 ppm Ethanol	[18]
Porous silicon/gold nanostructures	JFET (junction-field-effect transistor)	NO_2_	*ΔI*/*I*_0_ = 0.64 (500 ppb NO_2_) *T* = Room temperature	[22]
YCoO_3_ perovskite	Conductometric	CO, NO_2_, NO, CH_4_	*T* = 160–200 °C	[24]
SiOCN/CNT	Conductometric	NH_3_, NO_2_	*LOD* = 10 ppm NH_3_, 2 ppm NO_2_; T = 100–350 °C	[28]
Graphene/polystyrene-sulfonate (rGO/PSS)	Conductometric	TMA	LOD = 22.7 mg/L TMA	[29]
Graphene oxide	Conductometric	NO_2_	LOD = 22 ppb NO_2_	[33]
Pd NPs/graphene	Conductometric	H_2_	*[ΔG/G]*100 = 24 (1% H_2_)	[34]
Porphyrins, pH indicators blend	Colorimetric	Volatile compounds	NA	[35]
Porphyrins and corroles	Quartz microbalance	Volatile compounds	Classification of breath analysis and food samples	[36,38]
Graphene/polystyrene- sulfonate	Conductometric	Trimethylamine	LOD: 23 mg/L	[29]
Graphene/polyaniline	Conductometric	Limonene Ethanol	LOD 80 ppm/limonene LOD: 800 ppm ethanol	[30]
Reduced graphene oxide	Conductometric	CO_2_	LOD: 420 ppm	[32]

* LOD: limit of detection.

**Table 2:** Feature of some electrochemical sensors discussed herewith.

Electrode System	Analyte	Method **	LOD *	In Real Matrix	Ref.
Au nanoelectrode ensemble	As(III)	SSQWv	5 ng/L	Yes	[47]
CB + AuNP on SPE	Glucose, hydrogen peroxide, hydro-quinone, ascorbic acid	LSV	0.87, 0.18, 0.012, 0.021 nM, respectively	Simulated	[48]
Co/Al LDH	Glucose, fructose, galactose, xylose, ribose, sucrose, maltose, lactose	Chronoamperometry in FIA	0.01 to 0.05 mM	Yes	[43]
Graphene/ionic liquid	Caffeic Acid	Electrochemical measurement	0.005 mM	Simulated	[44]
ZnO nanorods coate by Co and Mn porphyrins	l-Cysteine	LSV	N.A.		[45]

* LOD values suffer from the different way how they are computed. Most often, the choice of estimating the signal leading to LOD as three times the standard deviation of the background leads to better or even much better results than the more correct calculation also considering the standard deviation of intercept and slope of the regression equation [39]. ** SSQWV: Stripping Square Wave Voltammetry; CB: carbon black; SQWV: square wave voltammetry; NP nanoparticles; LSV linear sweep voltammetry; FIA flow injection analysis.

**Table 3:** Characteristics of the catalytic biosensors discussed in the paper.

Target Analyte	Biological Element	Transducer Element	Method	Target Matrix	Ref.
Cholesterol	Cholesterol oxidase	Prussian Blue modified SPE	Amperometric	Human serum	[48]
Lactate	Lactate oxidase	Prussian Blue modified SPE	Amperometric	Wine	[49]
Polyamines	Polyamine oxidase, spermine oxidase	Prussian Blue modified SPE	Amperometric	Food	[50]
Lysine	Lysine oxidase	Pt electrode + overoxidised polypirrole	Amperometric	Cheese	[51]
Glucose	Glucose oxidase	Transdermal microneedles	Amperometric	Transdermal fluids	[52]
Glucose	Glucose oxidase	Gold nanoelectrode ensembles	Amperometric mediated	Not specified	[53]
Ethanol	Alcohol dehydrogenase	Polyaniline doped modified SPE	Amperometric	Wine	[54]
Antioxidant capacity	Superoxide dismutase	Pt electrode	Amperometric	Fruit juices and berries	[55]
Antioxidant capacity + ascorbate	Ascorbate oxidase	Fullerenes + nanotubes modified graphite	Amperometric, differential	Fruit juices	[56]
Atrazine	Tyrosinase	Different carbon modified SPE	Amperometric, inhibition	Drinking water	[57]
Oxygen profile	Biliribine oxidase	Pt electrode	Amperometric	Microbial fuel cell	[58]
Diuron, chlorpyrifos, catechol, urea, lactose, d-lactic acid.	Acetylcholinesterase, tyrosinase, urease, β-galactosidase, d-lactate dehydrogenase, *C. reinhardtii* cells	Custom made fluorimeter	Fluorescense of fluorescein 5(6)-isothiocyanate, 5(6)-carboxynaphtho-fluorescein or fluorescence emission of chlorophyll inhibition	Milk safety	[59]

**Table 4:** Summary table of affinity biosensors.

Target Analyte	Biological Element	Transducer Element	Method	Target Matrix	Ref.
IgG	Antibody	Titania-silica-coated long period gratings optical fibers	Label-free evanescent wave	Human serum	[60]
Prostate specific antigen	Antibody	Dense arrays of micropillars	Label-free CCD + software for imaging	Human serum	[61]
Interleukin 4	Antibody	Organic transistor	Label-free field effect transistor	Human serum	[62]
Cortisol	Antibody	Mobile phone	Lateral flow ELISA, chemiluminescent	Human saliva	[63]
Aflatoxin B1	Aptamer	Dendrimer- modified gold electrode	Voltammetric/impedimetric	Peanuts and peanuts corn snacks	[64]
Aflatoxin M1	Aptamer	Multiple SiON microring resonators	Label-free with silicon detector in microfluidics	Dairy products	[65]
Metalloproteinase 9	Aptamers	Quartz crystal microbalance	Label-free, piezoeletric using 2 different aptamers	Human serum	[66]
DNA methylation	Aptamers for α-thrombin	Au coated magnetic nanoparticles	Colorimetric, aggregation using 2 aptamers	DNA	[67]
Human epidermal growth factor receptor 2	Affibody	Au-nano-particles on SPE	Impedimetric	Human serum	[68]
Bicalutamide analogues	Human and rat serum albumin	Gold chip	Label free surface plasmon resonance	Not specified	[69]
Enantiomers of carvone	Odorant binding proteins	Organic transistor	Field effect transsistor in gas phase	Not specified	[70]
Octenol and carvones	Bovine and porcine odorant binding proteins	Array of five resonators coated with different proteins	Surface acoustic wave in gas phase	food	[71]
Butanal	Porcine odorant binding protein	Plastic fibers	Surface plasmon resonance	Food	[72]
Off-flavours in chocolate	Short peptide sequences on AuNPs	Array of seven quartz crystals coated with different peptides	Piezolectric in gas phase	Chocolate	[73]
Human DNA topoisomerase IB activity		Graphene monolayer	Field-effect	Not specified	[74]
Human genomic DNA sequences	DNA	Gold chips platform	Surface plasmon resonance imaging with Au nanostar- labelled complentary strands	Human genomic DNA	[75]
Roundup Ready soy gene	Peptide nucleic acid	Microstructured optical fibers	Optical detection with spreptavidin coated AuNPs	Soy	[76]
Tuberculosis mycobacterium	Peptide nucleic acid	Grating coupled surface resonators	Surface plasmon resonance	Not specified	[77]
7 kbps clone	DNA	Quartz crystal microbalance	Piezoeletric	Not specified	[78]
Single nucleotide mutation in p53 tumor suppressor gene	DNA	Carbon based SPE	Amperometric, biotin-avidin enzyme amplified	Not specified	[79]
